# Two 8-Hydroxyquinolinate Based Supramolecular Coordination Compounds: Synthesis, Structures and Spectral Properties

**DOI:** 10.3390/ma10030313

**Published:** 2017-03-18

**Authors:** Chengfeng Zhu, Yunfei Wang, Qingqing Mao, Fang Li, Yougui Li, Changle Chen

**Affiliations:** 1 School of Chemistry and Chemical Engineering, Hefei University of Technology, Hefei 230009, China; wangyunfeihfut@163.com (Y.W.); 18242311152@163.com (Q.M.); lifang@mail.ustc.edu.cn (F.L.); liyg1224@126.com (Y.L.); 2 Department of Polymer Science and Engineering, University of Science & Technology of China, Jinzhai Rd. 96, Hefei 230026, China

**Keywords:** 8-hydroxyquinoline, chromium(III) complex, supramolecular coordination compound

## Abstract

Two new Cr(III) complexes based on 2-substituted 8-hydroxyquinoline ligands, namely [Cr(**L**_1_)_3_] (**1**), (H**L_1_**=(*E*)-2-[2-(4-nitro-phenyl)-vinyl]-8-hydroxy-quinoline) and [Cr(**L**_2_)_3_] (**2**), (H**L_2_**=(*E*)-2-[2-(4-chloro-phenyl)vinyl]-8-hydroxy-quinoline), were prepared by a facile hydrothermal method and characterized thoroughly by single crystal X-ray diffraction, powder X-ray diffraction, FTIR, TGA, ESI-MS, UV-Visible absorption spectra and fluorescence emission spectra. Single crystal X-ray diffraction analyses showed that the two compounds featured 3D supramolecular architectures constructed from noncovalent interactions, such as π···π stacking, C-H···π, C-H···O, C-Cl···π, C-H···Cl interactions. The thermogravimetric analysis and ESI-MS study of compounds **1** and **2** suggested that the Cr(III) complexes possessed good stability both in solid and solution. In addition, the ultraviolet and fluorescence response of the H**L_1_** and H**L_2_** shown marked changes upon their complexation with Cr(III) ion, which indicated that the two 8-hydroxyquinolinate based ligand are promising heavy metal chelating agent for Cr^3+^.

## 1. Introduction

Complexes of various metals with 8-hydroxyquinoline (HQ) or its derivatives have attracted considerable attention owning to their biological activities and promising application in organic light-emitting diodes (OLEDs), optical sensing, and so on [1,2,3,4,5,6]. In recent years, particular interest has been given to the supramolecular coordination compounds based on 8-hydroxyquinoline derivatives [7,8,9,10,11]. Because of the ease of preparation and chemical modification of 8-HQ, various 8-hydroxyquinoline derivatives with different substituents are employed to construct metal complexes with desired structure and properties relying on the non-covalent intra- and intermolecular forces [12,13,14]. For example, Yuan et al. recently reported that the luminescent properties of trimeric Zn(II) 8-hydroxyquinolinates tuned by functional substituents [8]. In addition, a wide range of metal ions, including main group, transition, and rare earth metal ions, have been used in preparing 8-hydroxyquinolinates-based supramolecular coordination compounds with the aim to understand the effect of the central metals on supramolecular architectures as well as the properties of the final products [15,16,17,18,19,20,21].

It is known that Cr(III) and Al(III) have very similar ionic radii, coordination numbers (typically six) as well as comparable thermodynamic stabilities. In addition, 8-hydroxyquinolinate complexes of Cr(III) and Al(III) also exhibit excellent electronic spectral properties [22,23,24,25]. However, to the best of our knowledge, Cr(III) 8-hydroxyquinolates are rarely explored, even though 8-hydroxyquinoline shown excellent coordination ability with various metal ions [26,27]. The difficulty in synthesis of Cr(III) 8-hydroxyquinolate might arise from the high kinetic inertness of Cr^3+^ ion with the 3 *d*^3^ electronic configuration. In order to gain valuable information on both the structure and spectral properties of Cr(III) 8-hydroxyquinolate and investigate their promising application, we report here the synthesis and characterization studies of two Cr(III)-based supramolecular coordination compounds constructed from 2-substituted 8-hydroxyquinoline ligands.

## 2. Materials and Methods

### 2.1. General

All of the chemicals are commercial available, and used without further purification. Thermogravimetric analyses (TGA) were carried out at a rate of 10 °C/min in the temperature range of 50–800 °C under a nitrogen atmosphere on a STA449C integration thermal analyzer (Netzsch, Bavaria, Germany). The infrared spectra (KBr pellet) were recorded (400–4000 cm^−1^ region) on a Nicolet Magna 750 FT-IR spectrometer (Nicolet Instrument Corporation, Markham, ON, Canada). Electrospray ionization mass spectra (ES-MS) were recorded on a MALDI-TOF mass spectrometer (Bruker Daltonics Inc., Bremen, Germany) using dichloromethane-methanol as mobile phase. All UV-Vis absorption spectra were recorded on a Lambda 20 UV-Vis Spectrometer (Perkin Elmer, Inc., Waltham, MA, USA). The fluorescence spectra were carried out on a LS 50B Luminescence Spectrometer (Perkin Elmer, Inc., Beaconsfiled, Backinghamshire, UK). The EPR spectra were acquired on JES-FA200 electron paramagnetic resonance spectrometer (JEOL, Tokyo, Japan) at room temperature on X-band 9.07 GHz frequency under the magnetic field strength of 337 G.

### 2.2. Synthesis of ***1***

Cr(OAc)_3_ (2.29 mg, 0.01 mmol) and H**L_1_** (8.76 mg, 0.03 mmol) were dissolved in a DMSO/H_2_O/EtOH (3 mL/0.2 mL/3 mL) in a 20 mL vial. Then the mixture were heat at 80 °C for 24 h, 6.48 mg reddish brown crystals of **1** were obtained with a yield of ~70% based on Cr.

### 2.3. Synthesis of ***2***

Using the same procedure as that used for **1**, except using H**L_2_** (8.43 mg, 0.03 mmol) instead of H**L_1_**, brown block crystals of **2** were obtained with a yield of ~62.3% based on Cr.

### 2.4. X-ray Crystallography

Single-crystal XRD data for compounds **1** and **2** were collected on a Bruker SMART Apex II CCD-based X-ray diffractometer (Bruker AXS GmbH, Karlsruhe, Germany) with Mo-Kα radiation (λ = 0.7103 Å) at 296 K, respectively. The structures of compounds **1** and **2** were solved using direct method, and refined by full-matrix least-squares on F^2^, Technical details of the crystal structure solutions and refinements are listed in Table 1. Powder X-ray diffraction (PXRD) data were collected on a DMAX2500 diffractometer (Rigaku, Tokyo, Japan) using Cu Kα radiation. The calculated PXRD patterns were produced using the SHELXTL-XPOW program (Version 5.102, Bruker AXS, Germany) and single crystal reflection data.

## 3. Results and Discussion

### 3.1. Single Crystal Structures

**Scheme 1:**
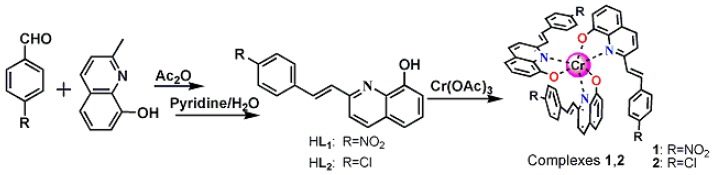
Synthesis of **1** and **2**.

**Figure 1:**
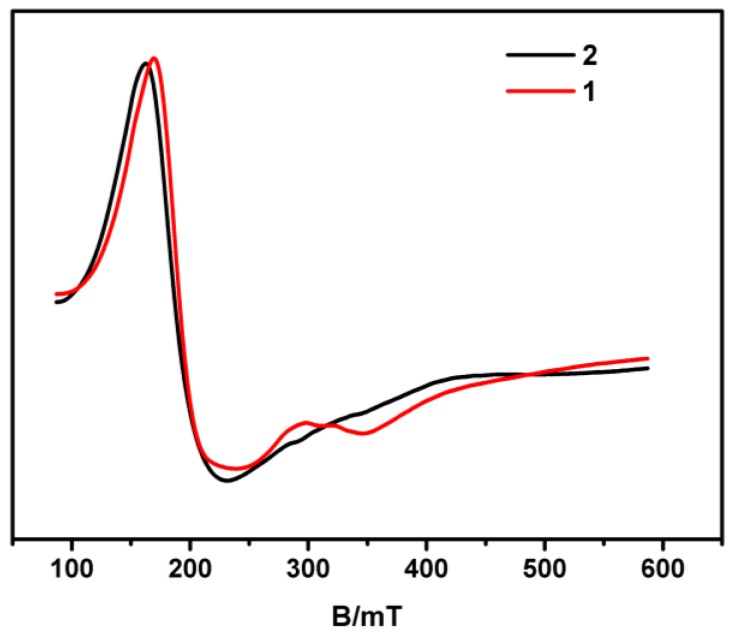
Electron paramagnetic resonance (EPR) spectra of the crystalline samples of complex **1** and **2** at room temperature and v = 9.07 GHz. Broad intense transitions are observed at 168 and 162 mT resonance fields, respectively.

**Figure 2:**
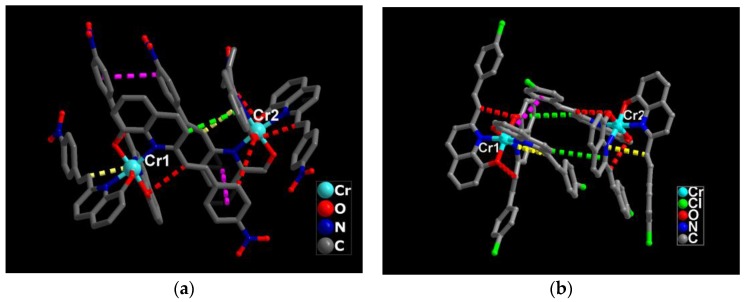
Views of the coordination geometries of Cr(III) atoms and the noncovalent interactions in the mononuclear complexes **1** (**a**) and **2** (**b**) (The intermolecular interactions are shown in different colors for clarity, the C-H···O, C-H···N, C-H···π and π···π stacking interactions were highlighted by red, yellow, green, and pink dotted lines, respectively).

As shown in Scheme 1, two 2-substituted 8-hydroxyquinoline ligands were readily synthesized according the reported procedure [14,28]. A solvothermal reaction of Cr(OAc)_3_ with H**L_1_** or H**L_2_** (1:3 molar ratio) in DMSO/H_2_O/EtOH afforded brown crystals of Cr(**L**_1_)_3_ (**1**) or Cr(**L**_2_)_3_ (**2**) in good yields. A single-crystal X-ray diffraction study reveals that **1** crystallizes in the triclinic space group *P*-1, with two crystalgraphic independent Cr(**L_1_**)_3_ molecules in the asymmetric unit. Three 8-hydroxyquinolinate ligands **L_1_** chelate to one Cr centers in the *mer*-geometry to form a propeller-like Cr(**L_1_**)_3_, thus, the Cr centers adopt an octahedral coordination environment, which was confirmed by the paramagnetic *S* = 3/2 system (*d*^3^ electron configuration) of Cr center in complex **1** (see Figure 1) [29,30]. In the octahedral geometry, the Cr-O, Cr-N distances are fall in the range of 1.9176(2)–1.949(2) Å and 2.102(2)–2.186(2) Å, respectively (See Table 2), which are comparable with those reported [26]. The three 8-hydroxyquinolinolates are almost perpendicular to each other, with dihedral angles of 85.7, 86.90, 87.31° for Cr_1_(**L_1_**)_3_ unit and 87.55, 89.10, 86.35° for Cr_2_(**L_1_**)_3_ unit, respectively. As shown in Figure 2a, multiple intramolecular C-H···O and C-H···N hydrogen bonds could be found between the C-H group of ethenyl and quinoline rings (for Cr_1_**L_1_**, C···O = 3.063(4)–3.153(4) Å, C-H···O = 136.0°–152.0°; for Cr_2_**L_1_**, C···O = 2.945(4)–2.992(4) Å, C-H···O = 131.0°–137.0°, C···N = 3.183(4) Å, C-H···N = 135°), further stabilizing the whole motif of mononuclear Cr(**L_1_**)_3_ units.

**Figure 3:**
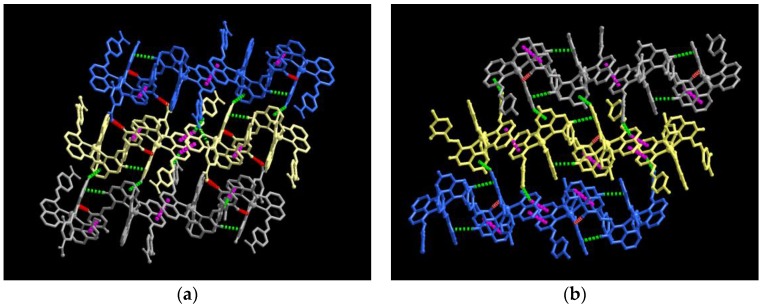
Views of the two-dimensional supramolecular structure of **1** (**a**) and **2** (**b**). (The 1D chains are shown in different colors for clarity, the C-H···O, C-H···π and π···π stacking interactions were highlighted by red, green, and pink dotted lines, respectively).

**Figure 4:**
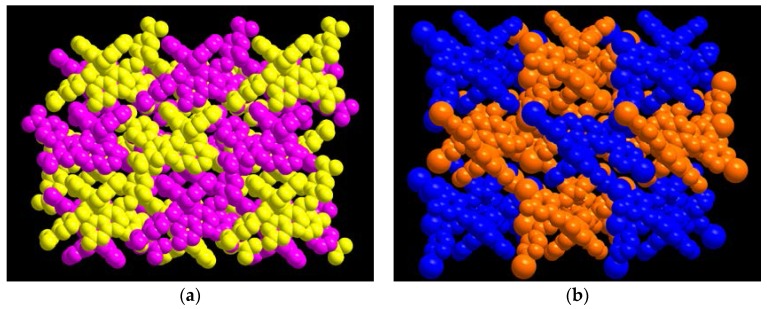
Views of the space-filling representations of the three-dimensional supramolecular structures of **1** (**a**) and **2** (**b**).

The two Cr(**L_1_**)_3_ entities in the asymmetric unit are linked together as a dimer by a bundle of supramolecular interaction with a Cr1-Cr2 distance of 8.979(1) Å, including by H-bonding interaction [C(3)-O(15) = 3.226(4) Å], C-H···π interaction [(C(10)-H(10)···π = 3.724(4) Å] and two π···π interactions (centroid-centroid distance is 3.804(6) Å between the adjacent 4-nitrophenyl rings; centroid-centroid distance is 3.779(4) Å between the 4-nitrophenyl ring and quinoline unit). Among the parallel quinoline rings from Cr(**L_1_**)_3_ units, the π^…^π stacking interactions (face-to-face distance: 3.612(5)–3.662(3) Å) direct the assembly of neighboring dimers into a 1D supramolecular chain (Figure 3a). The adjacent chains further aggregate into a 2D supramolecular layers in the *bc* plane via interchain H-bonding interactions [C(6)-O(13) = 3.365(5) Å, C(73)-O(8) = 3.359(7) Å] and interchain C-H···π interactions [C(84)-H(84)···π = 3.527(4) Å; C(22)-H(22)···π = 3.554(4) Å]. Stacking of 2D layers along the crystallographic *a*-axis assisted by C-H···π interaction [C(53)-H(53)···π = 3.853(5) Å] finally results in a 3D supramolecular architecture (Figure 3). The potential solvent-accessible volume for **1** is calculated using Platon, and it suggests void space only constitutes 2.4% of the total crystal volume, i.e., **1** is nearly a non-porous solid (Figure 4a) [31].

To compare the influence of different substituents at 2-position on the supramolecular self-assembly, ligand H**L_2_** was employed to prepare supramolecular coordination compound **2** [12,32]. Compound **2** crystallizes in space group *P*-1 and is isostructural with compound **1**, but the arrangement of building units (Cr(**L_2_**)_3_ molecules) in the supramolecular network is different due to the non-covalent interactions originated from Cl. As depicted in Figure 2b, each of Cr(III) ions in **2** adopts a distorted octahedral geometry constructed from three oxygen and three nitrogen atoms of three L**_2_** ligands. The bond lengths around Cr centers are similar with those in **1** (see Table 1). The two crystallographically unique Cr(**L_2_**)_3_ molecules in the asymmetric unit with a Cr1-Cr2 separation of 8.853(1) Å are linked into a Cr_2_(**L_2_**)_6_ dimer via the combination of weak π···π stacking (face-to-face distance: 3.828(18) Å), H-bonding interaction [C(68)-O(2) = 3.311(4) Å] as well as intermolecular C-H···π interaction between adjacent quinoline units [C-H···π = 3.736(4), 3.870(4) Å, respectively. As seen in Figure 3b, the Cr_2_(**L_2_**)_6_ dimers connect with neighboring units in plane [1,0,0] to form a supramolecular 2D layer via π···π interactions of the adjacent quinoline units (centroid-centroid distances are in the range of 3.646(2)–3.679(2) Å) and intermolecular C-H···π interactions (C-H···π = 3.496(4), 3.445(4) Å), respectively. Unlike **1**, the construction of a 3D supramolecular network employing these 2D layers is assisted not only by the weak intermolecular C-H···π interactions [C(50)-H(50)···π = 3.710(7) Å] but also by the C-Cl···π interactions [C(100)-Cl···π = 3.654(3) Å] and C-H···Cl interactions (C(55)-H(55)···Cl(3) = 3.286(5) Å). This is primarily due to the replacement of -NO_2_ group by the chlorine atom on the bidentate chelate ligand, leading to different intermolecular interactions in the solid state [8,9]. In addition, compound **2** is almost non-porous supramolecular coordination polymers with only 2.1% of void space as calculated by PLATON (see Figure 4b) [31].

### 3.2. The stability of ***1*** and ***2***

**Figure 5:**
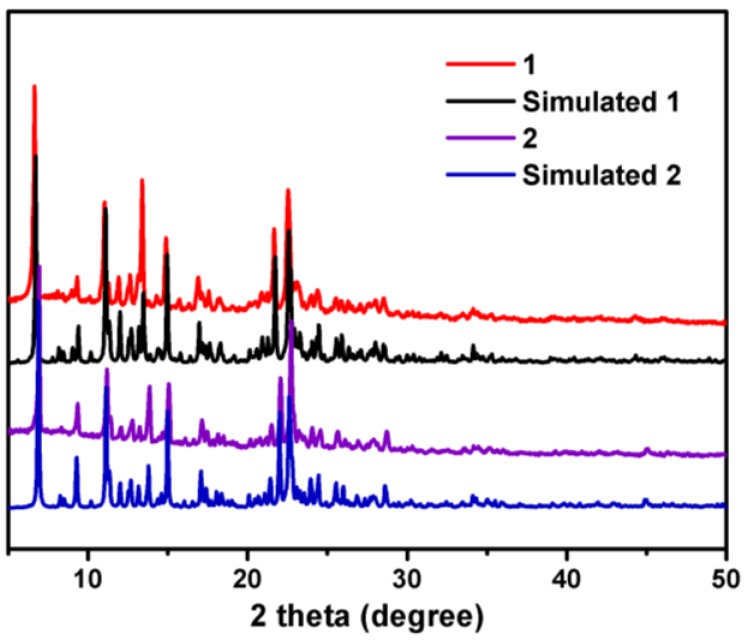
Powder X-ray diffraction (PXRD) patterns of complexes **1** and **2**.

**Figure 6:**
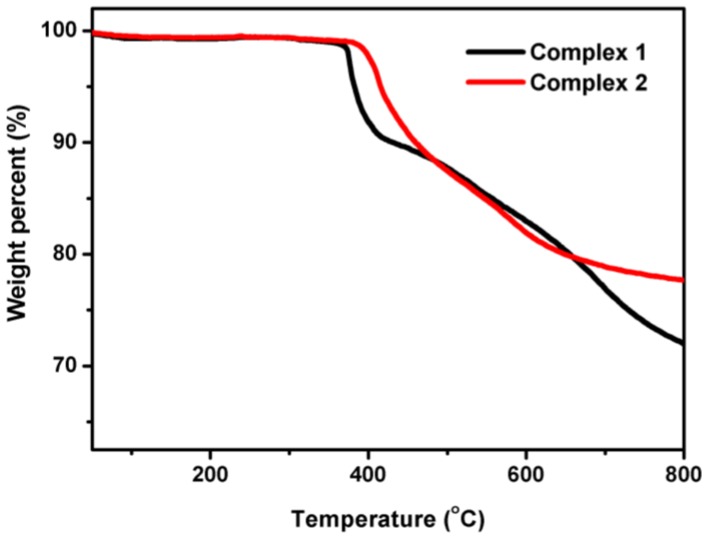
Thermogravimetric analysis (TGA) curves of complexes **1** and **2**.

In order to confirm the crystal structure of **1** and **2** are truly representative of their bulk samples, the PXRD experiments have been carried out on the as-prepared sample. As shown in Figure 5, the experimental diffraction patterns fit perfectly with the patterns simulated from their single-crystal structure, indicating the phase purity of bulk samples of **1** and **2**. The thermal stabilities of the two crystalline solids were determined by thermal analysis technique. Thermogravimetric analyses (TGA) of **1** and **2** shown no appreciable weight loss until the temperature reaches around 370 °C for **1** and 395 °C for **2**, respectively, this thermal behavior is similar to that of other metal quinolates [9,33] (see Figure 6). The TGA results suggest that the two supramolecular architectures are thermally robust, which are attributed to highly polarized Cr-N and Cr-O bonds. In comparison with compound **1**, the higher thermal stability may derive from C-Cl···π interactions and C-H···Cl interactions.

**Figure 7:**
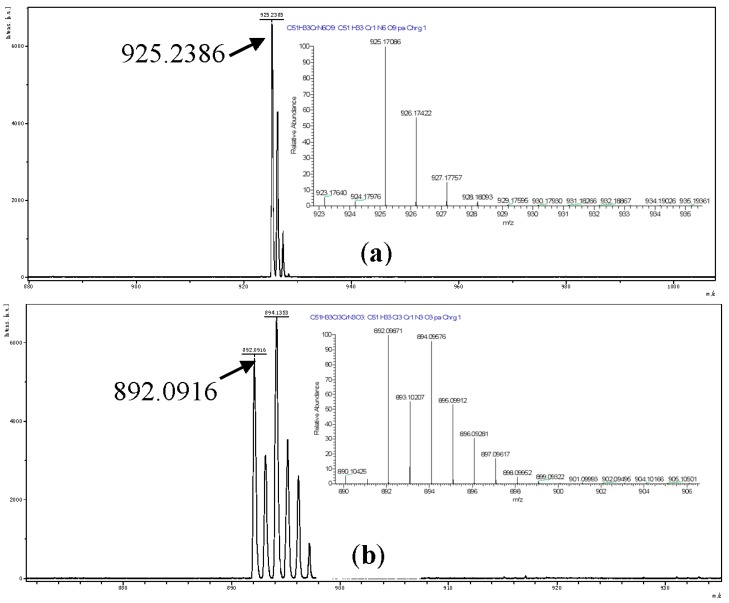
MALDI-TOF mass spectra of mononuclear complexes of Cr(**L_1_**)_3_ (**a**) and Cr(**L_2_**)_3_ (**b**), the insert pictures are the calculated MS spectra of **1** and **2**, respectively.

The stability of chromium(III) *tris*-(8-hydroxyquinolinates) in solution is confirmed from the electrospray ionization mass spectra of **1** and **2**, where the molecular ion peaks located at *m/z* = 925.2 and 892.1 are observed, corresponding to [Cr(**L_1_)**_3_]^+^ (calculated M^+^ = 925.1708) and Cr(**L_2_)**_3_]^+^ (calculated M^+^ = 892.0987), respectively (see Figure 7). The robustness of **1** and **2** is mainly due to kinetically inert nature of Cr(III), making it quite slow to undergo ligand exchange [34].

### 3.3. The IR spectra of ***1*** and ***2***

**Figure 8:**
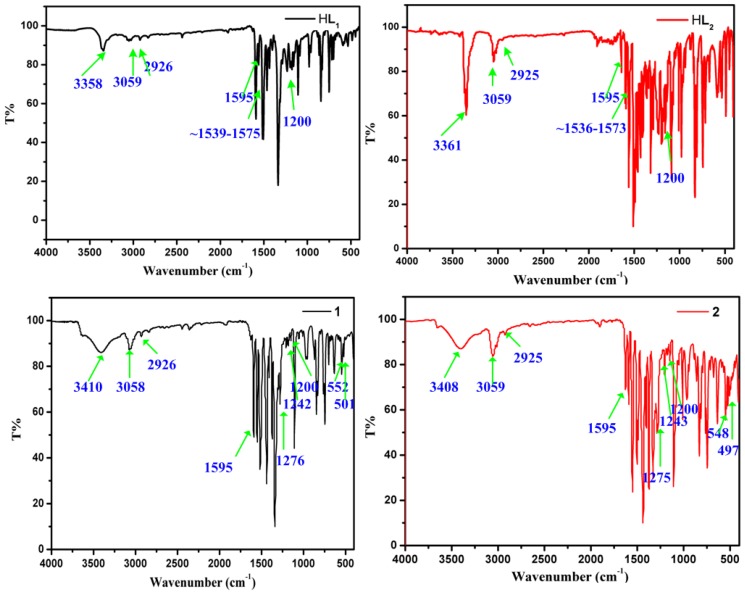
Infrared spectra of free ligands, complexes **1** and **2**.

The FT-IR spectra of the two bidentate ligands and corresponding Cr-based complexes are shown in Figure 8, and the proposed assignment of complexes are presented in Table 2. The characteristic ν (OH) stretching bands (located at ~3360 cm^−1^) and bending vibration bands (located at ~1200 cm^−1^) in the IR spectra of the two free ligand nearly disappears after coordination to Cr ion. The broad absorptions at ~3410 cm^−1^ in the spectra of two Cr(III) complexes are probably due to the presence of trace water in the KBr discs. The observation of an absorption bands located at ~3059 and 2925 cm^−1^ in the IR spectra of free ligands are attributed to the stretching vibrations of aromatic and vinylic C-H, which are here scarcely affected after complex formation. The ν (C=C) bands are found at similar energies as in the free ligands (~1536–1578 cm^−1^), respectively, but the ν (C=N) vibrational bands in the two Cr-based compounds are affected after complexation and exhibited at around ~1595 cm^−1^. After deprotonation, the band assigned to the ν (C-O) vibration moved to somewhat higher energies in the complexes (from 1205 to 1276 cm^−1^). In addition, the bands related to Cr-O and Cr-N vibrations are exhibited within region 552–497 cm^−1^ as refereed in Table 3 [35].

### 3.4. UV/Visible Absorption and Fluorescence Emission Spectra Studies

**Figure 9:**
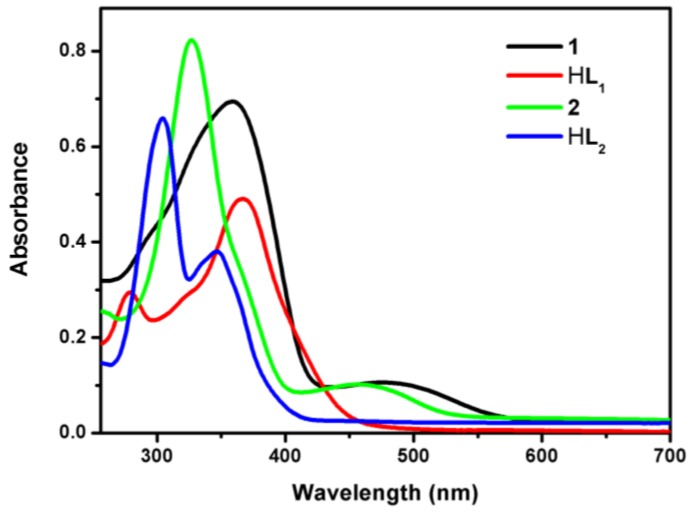
UV-Visible absorption spectra of H**L_1_**, H**L_2_**, **1**, and **2** in DMF solution (1.5 mM).

**Figure 10:**
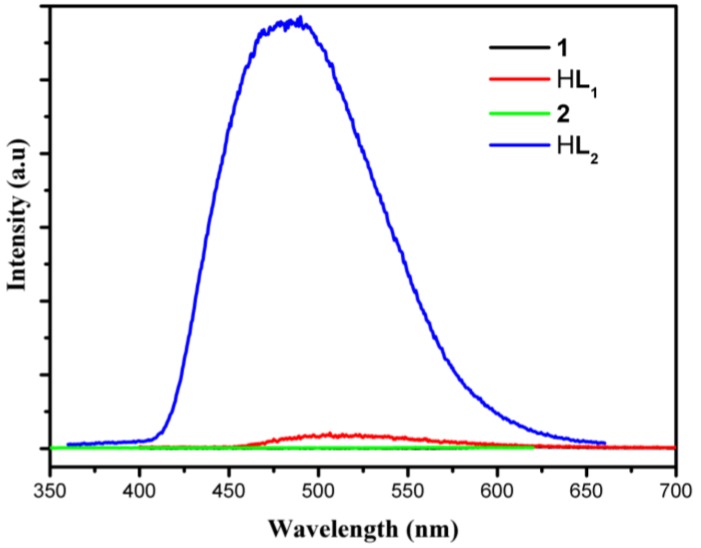
Emission spectra of H**L_1_**, H**L_2_**, **1**, and **2** in DMF solution (1.5 mM).

We obtained UV-vis spectra of H**L_1_**, H**L_2_**, **1**, and **2** in DMF (*N*,*N*-dimethylformamide) (see Figure 9). The absorption peaks around 275 nm in H**L_1_** and 305 nm in H**L_2_** can be assigned to the π-π* transition of the conjugated ligands, while the absorption peaks around 365 nm in H**L_1_** and 350 nm in H**L_2_** can be assigned to the n-π* transition of conjugated quinoline rings [35,36]. From the absorption spectra of **1** and **2**, we note that main characteristic absorption peaks corresponding to ligands exhibit red-shift. The main absorption bands of **1** and **2** merge into one broad peak centered at 360 nm and 325 nm, respectively, with a shoulder around 295 nm and 365 nm accordingly. Additionally, the emerging absorbance in the visible range around 480 nm in **1** and 460 nm in **2** might be ascribed to metal-to-ligand charge-transfer transitions (MLCT) [34,35]. The fluorescent properties of both the complexes and related 8-hydroxyquinolate ligands are also investigated in DMF at room temperature. Upon excitation at 365 nm, H**L_1_** and H**L_2_** display maximum emission wavelengths at 525 nm and 488 nm, respectively (see Figure 10). The very low emission of H**L_1_** in DMF solution is probably due to the strong electron withdrawing effect of nitro group on the aromatic ring [37,38]. However, the fluorescent emission of the 2-substituted 8-hydroxyquinoline ligands in DMF solution are completely quenched after the formation of Cr(III) hydroxyquinolinates (see Figure 10), which are similar to the result in the solid state (see below).

**Figure 11:**
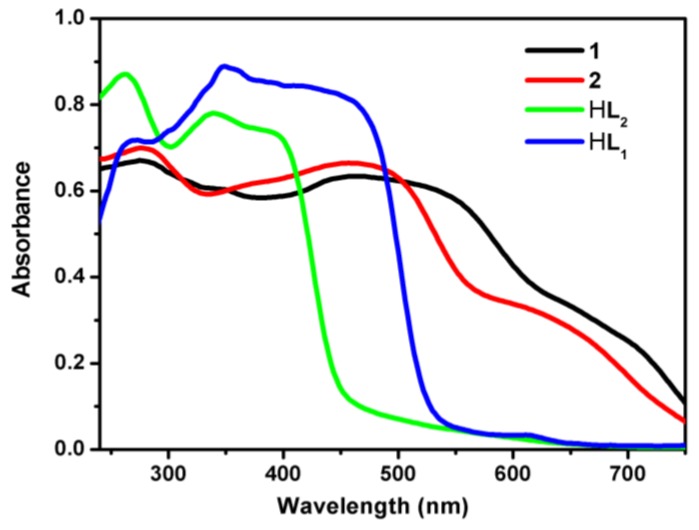
UV-Visible absorption of H**L_1_**, H**L_2_**, **1**, and **2** in solid state.

**Figure 12:**
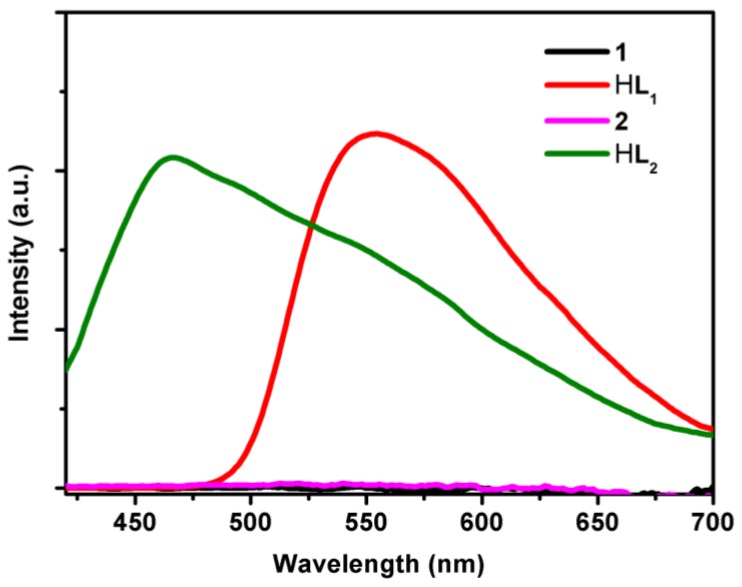
Emission spectra of H**L_1_**, H**L_2_**, **1**, and **2** in solid state.

The solid-state diffuse reflectance spectra of H**L_1_**, H**L_2_**, **1**, and **2** present similar bands to those observed in solution, albeit with a slight blue shift. The ligand H**L_1_** shows two broad bands around 269 nm and 350 nm, while H**L_2_** shows two broad bands centered at 262 nm and 340 nm (see Figure 11). All of these absorption bands could be ascribed to the π-π* and *n*-π* transition of the conjugated system of ligands. Upon binding to the Cr(III) ion, the two main absorption bands of the two free ligands exhibit a red shift by ~15 nm, which is due to the enhancement of rigidity after the formation of complexes. Special absorptions at 480 nm and 460 nm are found for compounds **1** and **2**, respectively. We assign these broad absorptions as metal-to-ligand charge transfer transitions (MLCT) between center mental ions and ligands. It should be noted that the broad shoulder at ca. 670 nm for **1** and 635 nm for **2** can be attributed to *d*-*d* transition of chromium ion [34]. The emission spectra of the two 8-hydroxyquinoline ligands and their metallocomplexes are examined in the solid state at room temperature as well. Upon excitation at 350 nm, the major emission peaks located at approximately 550 and 465 nm for H**L_1_** and H**L_2_**, respectively, dominate the fluorescence spectra (see Figure 12). Though the five-membered chelate rings formed between the NO donors and Cr(III) metal centers have increased the π-π* conjugation and conformational coplanarity of 8-hydroxyquinolinate, it is hard to detect the fluorescent signal of the two metallocomplexes. In contrast to other complexes of 8-hydroxyquinoline and its derivatives with metal ions (e.g., Al(III), Ga(III), Zn(II), or Cd(II)), the 3 *d*^3^ Cr(III) ion exhibits striking quenching effect, which might be attributed to the lowest energy transition being metal rather than ligand based [34,39]. The striking fluorescence response of the two 8-hydroxyquinolinate based ligand towards Cr^3+^ both in solid and solution indicated they could serve as a good heavy metal chelating agent for Cr^3+^ in a range of environmental monitoring and biomedical applications.

## 4. Conclusions

In summary, two supramolecular coordination compounds, [Cr(L1)3] (**1**) and [Cr(L2)3] (**2**), were prepared by a solvothermal reaction of Cr(OAc)_3_ with two 2-substituted-8-hydroxyquinolate ligands, respectively. Structures of compounds **1** and **2** were fully characterized by FTIR, MS, TGA, EPR, and single and powder X-ray diffraction. They also featured a 3D supramolecular architecture constructed from abundant noncovalent inter- and intramolecular forces. The remarkable change in the photoluminescent properties of the two 2-substituted 8-hydroxyquinoline ligands and their corresponding Cr-based complexes, both in solution and solid state, indicate that the two 8-hydroxyquinolate ligands are promising chelating agents in luminescence investigation Cr(III) ion [1,35,40]. Therefore, this study provides an idea to explore new 8-hydroxyquinolinate based ligands and related supramolecular coordination compounds for environmental monitoring and biomedical applications.

## Figures and Tables

**Table 1:** Crystal data and structure refinement for compounds **1** and **2**.

Identification Code	1	2
Empirical formula	C_102_H_66_Cr_2_N_12_O_18_	C_102_H_66_Cl_6_Cr_2_N_6_O_6_
Formula weight	1851.67	1788.31
Temperature (K)	296(2) K	296(2) K
Crystal system	triclinic	triclinic
Space group	P-1	P-1
Unit cell dimensions	*a* = 13.1901(16) Å	*a* = 12.884(3) Å
	*b* = 16.133(2) Å	*b* = 16.175(3) Å
	*c* = 20.623(3) Å	*c* = 20.794(4) Å
	α = 77.999(2)°	α = 77.349(3)°
	β = 86.580(2)°	β = 87.155(2)°
	γ = 85.524(2)°	γ = 84.676(3)°
Volume (Å^3^), Z	4275.2(1), 2	4208.1(2), 2
Density (calculated) (mg/m^3^)	1.438	1.411
F(000)	1908	1836
Limiting indices	−17≤ *h* ≤ 16, −20 ≤ *k* ≤ 21, −18 ≤ *l* ≤ 26	−15 ≤ *h* ≤ 15, −19 ≤ *k* ≤ 17, −21 ≤ *l* ≤24
Reflections collected	27000	21571
Independent reflections	19127 [R(int) = 0.0261]	14500 [R(int) = 0.0271]
Completeness to theta	25.00/98.2%	25.00/97.7%
Data/restraints/parameters	19127/0/1207	14500/3/1119
Goodness-of-fit on *F*^2^	0.970	1.034
Final R indices [I > 2sigma(I)]	*R*1 = 0.0570, *wR*2 = 0.1379	*R*1 = 0.0497, *wR*2 = 0.1224
R indices (all data)	*R*1 = 0.1197, *wR*2 = 0.1707	*R*1 = 0.0858, *wR*2 = 0.1418
Largest diff. peak and hole (e/Å^−3^)	0.339 and −0.302	0.405 and −0.307

**Table 2:** Selected Bond length for **1** and **2**.

Complex 1				Complex 2			
Bond	Dist.	Bond	Dist.	Bond	Dist.	Bond	Dist.
Cr(1)-O(9)	1.9176(2)	Cr(1)-N(1)	2.130(2)	Cr(1)-O(3)	1.916(2)	Cr(1)-N(1)	2.122(2)
Cr(1)-O(3)	1.938(2)	Cr(1)-N(3)	2.172(2)	Cr(1)-O(1)	1.934(2)	Cr(1)-N(3)	2.133(2)
Cr(1)-O(6)	1.949(2)	Cr(1)-N(5)	2.102(2)	Cr(1)-O(2)	1.945(2)	Cr(1)-N(2)	2.196(3)
Cr(2)-O(12)	1.920(2)	Cr(2)-N(11)	2.121(2)	Cr(2)-O(6)	1.918(2)	Cr(2)-N(6)	2.098(2)
Cr(2)-O(18)	1.943(2)	Cr(2)-N(7)	2.140(2)	Cr(2)-O(4)	1.935(2)	Cr(2)-N(4)	2.135(2)
Cr(2)-O(15)	1.944(2)	Cr(2)-N(9)	2.186(2)	Cr(2)-O(5)	1.958(2)	Cr(2)-N(5)	2.175(2)

**Table 3:** Infrared spectral bands and assignments of two Cr-based complexes.

1	2	Assignments
3410	3408	ν (trace H_2_O in the KBr discs)
3058, 2926	3059, 2925	ν (aromatic and vinylic C-H)
1595	1593	ν (C=N)
1539~1575	1536~1573	ν (C=C)
1276, 1242, 1208	1275, 1243, 1205	ν (C-O)
548	552	ν (Cr-O)
497	501	ν (Cr-N)

## Supplementary Files

Supplementary File 1

## Appendix A

X-ray Crystallographic data for the structures reported in this paper has been deposited with the Cambridge Crystallographic Data Centre as supplementary publication NO. CCDC-1507348, CCDC-1507355 for compound 1 and 2, respectively. These data can be obtained free of charge from the Cambridge Crystallographic Data Centre via http://www.ccdc.cam.ac.uk/conts/retrieving.html.
